# Insulin Facilitates the Recovery of Myocardial Contractility and Conduction during Cardiac Compression in Rabbits with Bupivacaine-Induced Cardiovascular Collapse

**DOI:** 10.1155/2012/878764

**Published:** 2012-04-11

**Authors:** Solmon Yang, Tserendorj Uugangerel, In-ki Jang, Hyung-chul Lee, Jong Min Kim, Byeong-Cheol Kang, Chong Soo Kim, Kook-Hyun Lee

**Affiliations:** ^1^Department of Anesthesiology, Seoul National University Hospital, No. 103, Daehang-no, Jongno-gu, Seoul 110-744, Republic of Korea; ^2^Clinical Research Institute, Seoul National University Hospital, No. 103, Daehang-no, Jongno-gu, Seoul 110-744, Republic of Korea; ^3^Department of Anesthesiology, Boramae City Hospital, 20 Boramae-ro 5-Gil, Dongjak-gu, Seoul 156-707, Republic of Korea

## Abstract

Bupivacaine inhibits cardiac conduction and contractility. Insulin enhances cardiac repolarization and myocardial contractility. We hypothesizes that insulin therapy would be effective in resuscitating bupivacaine-induced cardiac toxicity in rabbits. Twelve rabbits were tracheally intubated and midline sternotomy was performed under general anesthesia. Cardiovascular collapse (CVC) was induced by an IV bolus injection of bupivacaine 10 mg/kg. The rabbits were treated with either saline (control) or insulin injection, administered as a 2 U/kg bolus. Internal cardiac massage was performed until the return of spontaneous circulation (ROSC) and the time to the return of sinus rhythm (ROSR) was also noted in both groups. Arterial blood pressure, and electrocardiography were continuously monitored for 30 min and plasma bupivacaine concentrations at every 5 min. The ROSC, ROSR and normalization of QRS duration were attained faster in the insulin-treated group than in the control group. At the ROSC, there was a significant difference in bupivacaine concentration between two groups. Insulin facilitates the return of myocardial contractility and conduction from bupivacaine-induced CVC in rabbits. However, recovery of cardiac conduction is dependent mainly on the change of plasma bupivacaine concentrations.

## 1. Introduction

Resuscitation following bupivacaine-induced cardiovascular collapse (CVC) is difficult and often resistant to conventional treatment [1]. Bupivacaine blocks cardiac Na^+^ channel [2, 3] and transient outward K+ currents [4]. Increased ECG intervals [5] and decreased R-wave amplitude in lead II [6] are shown by the bupivacaine infusion. Bupivacaine also decreases the maximal rate of depolarization in Purkinje fibers [7]. Alteration of Ca^++^ recruitment from sarcoplasmic reticulum [8] and mitochondrial ATP production [9] are related to the deterioration of myocardial contractility by bupivacaine.

Previous experiments have shown that insulin might be effective in reversing bupivacaine-induced cardiac depression probably by enhancing transient outward K^+^ current, Ca^++^ transport activity of sarcoplasmic reticulum, and improving myocardial energetics [5, 10]. In contrast, Stehr et al. demonstrate that insulin has a significant positive inotropic effect without affecting electrophysiologic parameters at a fixed bupivacaine concentration in an in vitro study [11].

Because bupivacaine impairs ventricular conduction in a dose-dependent manner and QRS widening has been shown as a function of bupivacaine concentration in an isolated rabbit heart model [12], we assume that insulin might facilitate myocardial contractility and conduction during resuscitation of bupivacaine-induced CVC in vivo. The purpose of this study was to generate CVC by a bolus injection of bupivacaine in rabbits and evaluate the effect of insulin treatment with an internal cardiac massage on the recovery of the hemodynamic and electrocardiographic variables while measuring plasma bupivacaine concentration.

## 2. Materials and Methods

This study was approved by the Institutional Animal Care and Use Committee of Seoul National University Hospital (07-0167). Twelve New Zealand white rabbits (about 3 kg) were sedated with ketamine 15 mg/kg and xylazine 10 mg/kg intramuscularly. A 22-gauge venous catheter was placed into the marginal ear vein. The trachea was intubated with a 3.0 mm cuffed tube; thereafter, vecuronium 0.2 mg/kg was injected IV, followed by 0.02 mg/kg at 30 min intervals. The animals were placed in a supine position and mechanical ventilation was accomplished with a Servo 900C ventilator (SIEMENS, Erlangen, Germany) to maintain normocarbia at a fraction of inspired oxygen (F_I_O_2_) of 0.3. Anesthesia was maintained with a continuous infusion of sodium pentobarbital 5 mg/kg/hr. Ringer's lactate solution was infused at a rate of 10 mL/kg/hr. Rectal temperature was maintained at 38-39°C with a heating pad. The carotid artery was cannulated for arterial pressure monitoring and blood gas sampling and the jugular vein for central venous pressure (CVP) monitoring and drug administration. Lead II of the electrocardiogram (ECG) was recorded continuously. The heart was exposed through the midline sternotomy for internal cardiac massage.

Each rabbit received a bolus infusion of 10 mg/kg bupivacaine over 5 seconds. The time was measured at the onset of criteria for CVC (heart rate (HR) <65 and mean arterial pressure (MAP) below 20 mmHg), when the rabbits were randomly assigned to two groups: control (C) and insulin (I) groups (*n* = 6 in each group). At CVC, pentobarbital infusion was discontinued and F_I_O_2_ was elevated to 1.0. Internal cardiac massage was immediately accomplished at a rate of more than 150/min to keep MAP higher than 40 mmHg. An IV bolus of 0.9% normal saline 2 mL/kg was given to the C group and regular insulin 2 U/kg (1 U/mL) to the I group. Sodium bicarbonate was administered as needed to maintain an arterial pH value of 7.35–7.45.

The elapsed time till the return of spontaneous contraction (ROSC) was defined when MAP over 40 mmHg was maintained longer than 1 min without cardiac massage. The return of sinus rhythm (ROSR) after CVC was also measured. MAP, HR, CVP, and ECG were continuously monitored and recorded at 1-minute interval for 30 min after CVC. An ECG analysis system (MAC 8 Marquette, Los Angeles, CA, USA) was used to measure QRS duration every 5 min and at the time of ROSC and ROSR.

Coronary perfusion pressure (CPP) was estimated by the arterial pressure-to-CVP gradient during the relaxation phases of cardiac contraction. Arterial blood samples were collected for gas analysis and measurement of serum Na^+^, K^+^, Ca^++^, glucose at baseline, at CVC, and every 5 min for 30 min, and plasma bupivacaine concentrations were also measured at CVC, ROSC, and ROSR as well as every 5 min.

At the end of each experiment, the rabbits were killed with KCL 10 mEq IV. Blood samples for a bupivacaine concentration assay were centrifuged at 2500 rpm for 20 min, and the plasma was stored at −50°C until analyzed by high-performance liquid chromatography.

Data were expressed as mean ± SD. The differences between two groups were identified with two-way analysis of variance. Changes over time within each group were evaluated by using analysis of variance for repeated measure with Bonferroni correction. SPSS (12.0 K) was used for the statistical analysis. A value of *P* < 0.05 was considered statistically significant.

## 3. Results

The two groups were comparable with respect to baseline hemodynamic variables (Table 1) and hemoglobin concentration (11.4 ± 0.8 mg/dL of C group 10.4 ± 1.4 mg/dL of I group). The time needed to CVC was 13 ± 6 sec in the C group, and 17 ± 12 sec in the I group. All animals survived by the internal cardiac massage delivered at the onset of CVC till the time of ROSC.

The ROSC was reached at 20 ± 5 min in the C group and at 8 ± 4 min (*P* < 0.05) in the I group. The ROSR appeared at 20 ± 6 min spontaneously after CVC in the C group and at 15 ± 3 min (*P* < 0.05) in the I group. At the ROSC, the plasma bupivacaine concentrations were 1.51 ± 0.37 ug/mL in the C group, and 2.31 ± 0.18 ug/mL (*P* < 0.05) in the I group. However, the bupivacaine concentrations on ROSR were 1.48 ± 0.28 ug/mL in the C group and 1.87 ± 0.26 ug/mL in the I group without significant difference.

The time course of HR, MAP, CPP, and bupivacaine concentration was similar in both groups for 30 min. QRS duration was prolonged on CVC and returned to baseline level at 30 min in the C group and at 20 min in the I group. At 15 min after CVC, QRS duration of the C group (171 ± 41 ms) was longer than that (71 ± 12 ms) of the I group (*P* < 0.05) (Table 1).

Arterial pH, PaO_2_, PaCO_2_, Na^+^, K^+^, Ca^++^, and glucose were comparable in both groups for 30 min (Table 2). PaO_2_ increased from 5 min at the F_I_O_2_ of 1.0 in both groups. PaCO_2_ was significantly low on CVC in both groups and maintained within normal range thereafter. K^+^ level of the I group decreased significantly after 10 min of insulin injection; however, it showed no significant between-group differences for 30 min. Blood glucose level of the I group decreased after 15 min without significant difference, compared with the C group.

**Figure 1:**
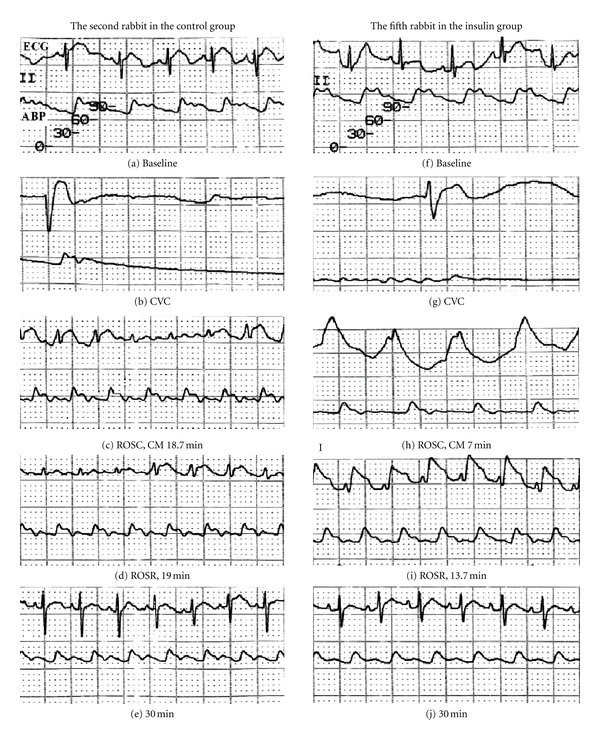
Sequential changes of ECG (upper) and ABP (lower) of the second rabbit of the control group (left column) and the fifth of the insulin group (right column), respectively. Lead II surface ECG was recorded at 25 mm/sec and 10 mm/mV. In the second rabbit of the control group, (a) during the baseline period, the values were; HR, 152 bpm, and QRS, 40 ms. (b) Bupivacaine injection caused sinus arrest and CVC. (c) At ROSC, CM stopped: HR, 205 bpm; QRS, 108 ms. (d) At ROSR: HR, 210 bpm; QRS, 82 ms. (e) At 30 min after CVC: HR, 212 bpm; QRS, 44 ms. In the fifth rabbit of the insulin group, (f) At baseline period: HR, 150 bpm; QRS, 48 ms. (g) Sinus arrest and CVC. (h) At ROSC, CM stopped: HR, 125 bpm; QRS, 200 ms. (i) At ROSR: HR, 225 bpm; QRS, 68 ms. (j) at 30 min after CVC: HR, 232 bpm; QRS, 48 ms. ECG: electrocardiogram; ABP: arterial blood pressure; bpm: beats per min; ms: millisecond; CVC: cardiovascular collapse; CM: cardiac massage; ROSC: return of spontaneous circulation; ROSR: return of sinus rhythm.

ECG changes and arterial blood pressure waves of an animal representing each group were shown in Figure 1. The lead II ECG of both animals showed sinus arrest on CVC when open cardiac compression was begun shortly thereafter. The second rabbit in the C group showed the ROSC and ROSR after 18.7 min and 19 min from CVC, and the fifth in the I group restored the ROSC and ROSR after 7 min and 13.7 min, respectively.

## 4. Discussion

We designed this study to observe the insulin effect on the recovery of cardiac hemodynamics and conduction during resuscitation after a bolus injection of bupivacaine in rabbits. The results showed that the recovery of ventricular contraction and conduction was enhanced after the insulin administration as compared with the control group during cardiac compression.

Bupivacaine blocks Na^+^ channel and thereby inhibits cardiac conductance by binding preferentially to the inactivated state of the sodium channel in ventricular muscle [3]. Bupivacaine also inhibits transient outward potassium current (I_to_) which is associated with myocardial repolarization [4] and depresses cardiac contractility by altering Ca^++^ release and Ca^++^ sequestration of the sarcoplasmic reticulum and decreases myofibrillar activation [13]. In contrast, insulin increases Ca^++^ transport activity of sarcoplasmic reticulum [14]. Transient outward K^+^ current is enhanced by insulin [15]. Since cardiac depressant effects of bupivacaine can be attenuated by shortening the action potential duration [3], I_to_ facilitated by insulin may promote depolarization, thus beneficial in reducing bupivacaine cardiotoxicity [4].

Bupivacaine was continuously infused in the previous studies using a dog model; thus cardiac depression was gradually induced [5, 10]. In this study, the effect of insulin was compared after bupivacaine was injected as a bolus dose and CVC was induced suddenly. About 15 seconds were taken to reach MAP below 20 mmHg and HR lower than 65/min after the bolus injection. In clinical situation, accidental administration of bupivacaine into systemic circulation may occur in a drastic manner, therefore, resulting in a severe cardiotoxicity which needs prompt resuscitation including cardiac massage.

Bupivacaine increases the QRS duration in a dose-dependent manner, while this impairment in ventricular conduction by bupivacaine is rate dependent [12]. Therefore, we tried to prove the insulin effect on the recovery of cardiac conduction in rabbits with higher baseline HR. In a pilot study, rabbits failed to recover spontaneously in the criteria of CVC (HR < 65/min and MBP below 20 mmHg) induced by a bolus injection of bupivacaine. In our experimental protocol, internal cardiac massage was continued till the time of ROSC and terminated thereafter. Myocardial blood flow is dependent on the aortic-to-right atrial pressure gradient during the relaxation phase of cardiac compression. CPP has been recommended more than 15 to 20 mmHg in cardiopulmonary resuscitation [16]. By the similar changes of MAP and CPP between two groups, we believe that internal cardiac massage till ROSC was effective in producing cardiac output for all animals.

The decrease in contractility measured by the *dP/dt *_max_ is linearly correlated with the bupivacaine concentration [12]. In this study, ROSC was reached faster with higher bupivacaine concentration in insulin-treated animals. The earlier recovery of arterial blood pressure at higher plasma bupivacaine level might be due to the positive inotropic effect of insulin [11]. As expected, ROSR was slower than ROSC because bupivacaine impairs ventricular conduction more than contractility [12].

ROSR appeared earlier in the insulin-treated group, but the bupivacaine concentrations on ROSR in both groups were not different significantly. This finding probably implies that insulin effect on cardiac conduction is mainly dependent on the change of plasma bupivacaine concentrations. Bupivacaine did not change cardiac automaticity but had a depressant effect on conduction at atrial, ventricular, and atrioventricular levels [17]. Thus, the sinus rhythm can be generated at a lower bupivacaine concentration where atrial electrical impulse can reach ventricular purkinje fibers via the atrioventricular node, resulting in the synchronous contraction of ventricular myocardium. However, faster recovery of QRS complex duration in the insulin-treated group reveals that ventricular conduction was enhanced by the insulin, resulting in the reduction of action potential duration [15].

Acidosis and hypoxia markedly potentiate cardiac toxicity [18]. Hence, F_I_O_2_ was changed from initial 0.3 to 1.0 at the onset of CVC, and sodium bicarbonate was infused to maintain arterial pH. The values of pH were maintained equally normal in both groups. Despite the effect of insulin on lowering plasma potassium levels, potassium was not added to the insulin-treated group. Hypokalemia can increase resting membrane potential and action potential height [19]. Thus, there might be a possibility that insulin-induced hypokalemia may enhance sodium inward current. However, hypokalemia may worsen toxicity by lengthening action potential duration [3], which was not believed to play a major role in reversing bupivacaine-induced cardiotoxicity [5]. Glucose was maintained above normal in both groups. The positive inotropic effects of insulin have been shown to be independent of the presence of glucose [11]. Despite of significant decrease in calcium level in both groups, serum calcium was maintained within the normal range throughout the experiment.

The limitation of this study is that experimental animals were mechanically ventilated to keep normocapnia with 100% oxygen and pH was controlled with sodium bicarbonate from the beginning of CVC. Hypoxia and hyperkalemia, known to increase bupivacaine's depressive effects [3, 20], were prevented. These might reduce the occurrence of severe arrhythmia such as ventricular tachycardia. Though this study was not blindly performed, internal cardiac massage was so effective that all animals survived. Because hemodynamics and bupivacaine concentration changes were similar between both groups, insulin effect on the recovery of cardiac contractility and conduction could be compared while minimizing the investigator bias. In spite of the fact that bupivacaine seems to render myocardium refractory to defibrillation [21], interaction or comparison of other treatment modalities with insulin therapy needs further investigations.

In conclusion, a single injection of insulin combined with open cardiac compression was helpful to restore spontaneous circulation and sinus rhythm after abrupt induction of CVC caused by a bolus injection of bupivacaine in rabbits. The result suggests that insulin therapy might be considered as a supplement to enhance cardiac contractility as well as to facilitate myocardial conduction for bupivacaine-induced cardiac toxicity.

## Figures and Tables

**Table 1:** Changes of hemodynamics, bupivacaine concentration and QRS duration in the control and insulin groups.

	Group	Baseline	CVC	5 min	10 min	15 min	20 min	25 min	30 min
HR (/min)	Control	184 ± 30	13 ± 30^†^	102 ± 14^†^	153 ± 21^†^	173 ± 25	215 ± 32	221 ± 31	219 ± 22
	Insulin	162 ± 36	21 ± 27^†^	109 ± 24^†^	150 ± 18	189 ± 31	185 ± 26	188 ± 31	188 ± 34

MAP (mmHg)	Control	88 ± 11	13 ± 4^†^	48 ± 9^†^	43 ± 6^†^	50 ± 15^†^	61 ± 25^†^	70 ± 22	84 ± 13
	Insulin	85 ± 15	13 ± 2^†^	44 ± 6^†^	36±7^†^	58±15^†^	72 ± 7^†^	73 ± 7	77 ± 9

CPP (mmHg)	Control	73 ± 12	6 ± 1^†^	23 ± 12^†^	20 ± 13^†^	29 ± 20^†^	48 ± 28	58 ± 21	73 ± 12
	Insulin	69 ± 13	7 ± 3^†^	20 ± 11^†^	19 ± 7^†^	43 ± 18^†^	58 ± 8^†^	61 ± 8	64 ± 10

Bupivacaine (ug/dL)	Control		84.89 ± 44.88^†^	3.21 ± 0.51^†^	2.02 ± 0.42^†^	1.59 ± 0.31^†^	1.41 ± 0.31^†^	1.21 ± 0.41^†^	1.09 ± 0.11^†^
	Insulin		77.45 ± 51.65^†^	3.42 ± 1.02^†^	2.21 ± 0.41^†^	1.81 ± 0.39^†^	1.62 ± 0.19^†^	1.49 ± 0.29^†^	1.19 ± 0.11^†^

QRS (msec)	Control	46 ± 9	168 ± 89^†^	178 ± 33^†^	173 ± 37^†^	171 ± 41^†^	75 ± 34^†^	65 ± 22^†^	55 ± 9
	Insulin	48 ± 9	186 ± 93^†^	182 ± 48^†^	176 ± 50^†^	71 ± 12^†,∗^	58 ± 12	58 ± 8	59 ± 9

Values are mean ± SD.

CVC: cardiovascular collapse; HR: heart rate; MAP: mean arterial pressure; CPP: coronary perfusion pressure.

*P < 0.05 versus control; ^†^ *P* < 0.05 versus baseline.

**Table 2:** Changes of arterial blood gas, serum electrolyte, and blood glucose concentration in the control and insulin groups.

	Group	Baseline	CVC	5 min	10 min	15 min	20 min	25 min	30 min
pH	Control	7.47 ± 0.03	7.50 ± 0.08	7.40 ± 0.06	7.40 ± 0.07	7.37 ± 0.09	7.36 ± 0.10	7.35 ± 0.10	7.37 ± 0.08
	Insulin	7.48 ± 0.07	7.51 ± 0.05	7.44 ± 0.06	7.45 ± 0.05	7.41 ± 0.05	7.41 ± 0.06	7.41 ± 0.06	7.42 ± 0.06

PaO_2_ (mmHg)	Control	139 ± 16	173 ± 33	353 ± 117^†^	330 ± 147^†^	379 ± 120^†^	370 ± 75^†^	340 ± 87^†^	333 ± 116^†^
	Insulin	137 ± 30	198 ± 98	351 ± 111^†^	430 ± 66^†^	399 ± 81^†^	397 ± 65^†^	401 ± 79^†^	426 ± 103^†^

PaCO_2_ (mmHg)	Control	34 ± 4	24 ± 5^†^	34 ± 3	35 ± 3	39 ± 8	42 ± 8	43 ± 9	42 ± 7
	Insulin	34 ± 4	27 ± 4^†^	29 ± 5	29 ± 5	37 ± 7	38 ± 6	38 ± 6	38 ± 6

Na^+^ (mEq/L)	Control	143 ± 4	143 ± 5	145 ± 5	145 ± 6	146 ± 6^†^	148 ± 6^†^	147 ± 5^†^	148 ± 5^†^
	Insulin	142 ± 3	142 ± 4	143 ± 3	144 ± 4	146 ± 4^†^	146 ± 3^†^	146 ± 3^†^	147±4^†^

K^+^ (mEq/L)	Control	2.9 ± 0.4	2.7 ± 0.5	2.8 ± 0.5	2.9 ± 0.6	3.0 ± 0.4	3.0 ± 0.5	3.0 ± 0.5	2.8 ± 0.5
	Insulin	3.0 ± 0.3	2.8 ± 0.4	2.8 ± 0.6	2.8 ± 0.6^†^	2.8 ± 0.5^†^	2.7 ± 0.4^†^	2.8 ± 0.4^†^	2.7 ± 0.3^†^

Ca^++^ (mEq/L)	Control	2.52 ± 0.21	1.98 ± 0.29^†^	2.08 ± 0.38^†^	2.11 ± 0.41^†^	1.99 ± 0.42^†^	2.03 ± 0.42^†^	2.02 ± 0.35^†^	2.08 ± 0.28^†^
	Insulin	2.79 ± 0.32	2.32 ± 0.21^†^	2.28 ± 0.29^†^	2.28 ± 0.29^†^	2.36 ± 0.23^†^	2.45 ± 0.24^†^	2.46 ± 0.22^†^	2.47 ± 0.18^†^

Glucose (mg/dL)	Control	256 ± 47	248 ± 50	261 ± 48	260 ± 63	242 ± 44	252 ± 71	255 ± 76	251 ± 74
	Insulin	262 ± 56	245 ± 70	230 ± 84	228 ± 90	197±55^†^	179 ± 53^†^	167 ± 54^†^	152 ± 56^†^

Values are mean ± SD.

CVC: cardiovascular collapse.

*P < 0.05 versus control; ^†^ *P* < 0.05 versus baseline.
